# A fast indirect method to compute functions of genomic relationships concerning genotyped and ungenotyped individuals, for diversity management

**DOI:** 10.1186/s12711-017-0363-9

**Published:** 2017-12-01

**Authors:** Jean-Jacques Colleau, Isabelle Palhière, Silvia T. Rodríguez-Ramilo, Andres Legarra

**Affiliations:** 1 GABI, INRA, AgroParisTech, Université Paris-Saclay, 78350 Jouy-en-Josas, France; 2 GenPhySE, Université de Toulouse, INRA, INPT, ENVT, Castanet Tolosan, France

## Abstract

**Background:**

Pedigree-based management of genetic diversity in populations, e.g., using optimal contributions, involves computation of the ${\mathbf{Ax}}$ type yielding elements (relationships) or functions (usually averages) of relationship matrices. For pedigree-based relationships ${\mathbf{A}}$, a very efficient method exists. When all the individuals of interest are genotyped, genomic management can be addressed using the genomic relationship matrix ${\mathbf{G}}$; however, to date, the computational problem of efficiently computing ${\mathbf{Gx}}$ has not been well studied. When some individuals of interest are not genotyped, genomic management should consider the relationship matrix ${\mathbf{H}}$ that combines genotyped and ungenotyped individuals; however, direct computation of ${\mathbf{Hx}}$ is computationally very demanding, because construction of a possibly huge matrix is required. Our work presents efficient ways of computing ${\mathbf{Gx}}$ and ${\mathbf{Hx}}$, with applications on real data from dairy sheep and dairy goat breeding schemes.

**Results:**

For genomic relationships, an efficient indirect computation with quadratic instead of cubic cost is ${\mathbf{x}} = {\mathbf{Z}}\left( {{\mathbf{Z^{\prime}x}}} \right)/k$, where **Z** is a matrix relating animals to genotypes. For the relationship matrix ${\mathbf{H}}$, we propose an indirect method based on the difference between vectors ${\mathbf{Hx}} - {\mathbf{Ax}}$, which involves computation of ${\mathbf{Ax}}$ and of products such as ${\mathbf{Gw}}$ and ${\mathbf{A}}_{22}^{ - 1} {\mathbf{w}}$, where ${\mathbf{w}}$ is a working vector derived from ${\mathbf{x}}$. The latter computation is the most demanding but can be done using sparse Cholesky decompositions of matrix ${\mathbf{A}}^{ - 1}$, which allows handling very large genomic and pedigree data files. Studies based on simulations reported in the literature show that the trends of average relationships in ${\mathbf{H}}$ and ${\mathbf{A}}$ differ as genomic selection proceeds. When selection is based on genomic relationships but management is based on pedigree data, the true genetic diversity is overestimated. However, our tests on real data from sheep and goat obtained before genomic selection started do not show this.

**Conclusions:**

We present efficient methods to compute elements and statistics of the genomic relationships ${\mathbf{G}}$ and of matrix ${\mathbf{H}}$ that combines ungenotyped and genotyped individuals. These methods should be useful to monitor and handle genomic diversity.

## Background

Optimal contribution [1–3] is a method of choice for the management of genomic diversity. In this method, reproducers are chosen such that the expected genetic gain and expected increase in homozygosity are properly weighted. The increase in homozygosity is estimated based on average relationships between selected individuals, and in livestock these relationships are usually pedigree-based. Such measures of diversity can be represented as ${\mathbf{x}}^{\prime } {\mathbf{Kx}}$ where ${\mathbf{K}}$ is a matrix of relationships and ${\mathbf{x}}$ a vector of contributions to the next generation. Optimizing contributions in ${\mathbf{x}}$ is a non-linear problem that requires repeated computation of ${\mathbf{x}}^{\prime } {\mathbf{Kx}}$, where the most difficult part is the computation of ${\mathbf{Kx}}$. In the case of pedigree relationships, a very fast method exists for this computation [4]. Here we recall that, in genomic selection, genomic relationships must be included in matrix ${\mathbf{K}}$ [5], and we present computational strategies in the case of genomic selection where all, or part of, the animals have been densely genotyped.

Genomic evaluation considers several tens of thousands of single nucleotide polymorphisms (SNPs) that are distributed across the whole genome, and in the most frequent implementation (genomic best linear unbiased prediction (GBLUP), or single-step GBLUP) it uses a so-called genomic relationship matrix. Following this approach, the accuracy in the evaluation of breeding values is improved compared to that of pedigree-based evaluation by exploiting existing linkage disequilibrium with neighboring quantitative trait loci (QTL) [6]. Consequently, genomic selection affects gene transmission, directly for SNPs and indirectly for QTL.

However, because of linkage between these high-density SNPs, indirect hitch-hiking also affects gene transmission at loci other than SNPs and QTL [7]. This fact impairs the conventional pedigree-based methods used for computing coancestry and inbreeding coefficients, where selection is assumed to be neutral, at any locus, concerning the gene transmission probabilities. Neutrality means that selection does not modify the probabilities of gene transmission within a given pedigree. For instance, before selection proceeds, the genotype of an unselected individual at a given locus comes from any possible grandparent pair with a probability of 1/4. However, when genomic selection occurs, grandparent combinations have local selective values that depend on the direction of selection (generally a combination of traits), and some of these combinations are better than others. Then, genomic selection restricts variability faster than predicted by the conventional algorithms based on pedigrees.

Sonesson et al. [5] illustrated by simulation that neutrality is impossible in a genomic selection scenario: they showed that the evolution of genomic relationship coefficients estimates the evolution of true inbreeding much more accurately than the evolution of pedigree-based coefficients. For instance, if two close sibs are selected because they have inherited the same beneficial allele at a QTL, and if they have a genomic relationship of 0.6, pedigree relationships can account for only 0.5 of the relationship. This is logical as genomic relationships describe *realized* instead of *expected* relationships, and take into account Mendelian segregation and linkage due to the finite size of the genome [8]. Thus, genomic management of genetic variability is required in order to avoid detrimental trends. For instance, Sonesson et al. [5] tried to maximize genetic gain when using genomic selection while restricting the rate of inbreeding per generation to 0.50% by using either genomic (each individual was genotyped) or pedigree-based coefficients. The true rate of inbreeding was 0.53% in the first case, which is in fairly good agreement with the restricted value. However, it reached a value as high as 2.26% when pedigree-based coefficients were used. In comparison, pedigree management with pedigree-based evaluation yields a true rate of inbreeding of only 0.74%, due to lower selection pressure on the QTL.

When monitoring evolution of genetic variability over time, or even optimizing management of genetic diversity at a given time, some individuals of interest may be ungenotyped (see “Appendix” for a comprehensive list of these situations). A simple example is when young genotyped rams are chosen, in which case these are genotyped whereas females are not. Estimating future inbreeding needs to consider both the genotyped rams and ungenotyped females.

Then, (ungenotyped, genotyped) and (ungenotyped, ungenotyped) relationships by combining pedigree and genomic information should be estimated. A natural approach is to use the matrix usually called ${\mathbf{H}}$ that was conceived to extend the information in genomic relationships to all individuals in a pedigree, regardless of the genotyping status [9]. Extensions of the theory accommodate different origins (metafounders), selection and drift [9–11]. Although it is most often used for genetic evaluation in the single-step GBLUP [9, 12], its use for management of diversity is natural, even if the evaluation is *not* by single-step GBLUP e.g., for dairy cattle where multi-step methods are the most common.

The objective of our study was to develop an indirect method for computing genomic relationship coefficients and vector functions ${\mathbf{Gx}}$ and ${\mathbf{Hx}}$, where the pedigree-based relationship matrix ${\mathbf{A}}$ may (or not) account for single or multiple origins. The method that we present here is useful to expedite the computations needed when monitoring or optimizing management of diversity in genomic selection, as already done by the indirect method for computing vectors ${\mathbf{Ax}}$ in the pedigree-based context [4].

The new approach was evaluated using data from dairy goat and dairy sheep breeding programs. We also discuss the issues that raise from using ${\mathbf{H}}$ instead of ${\mathbf{A}}$ in the world of practitioners and breeders and suggest methods to present genomic relationships at the classical pedigree scale via shift and scale conversion factors.

## Methods

### Computation of the matrix product ${\mathbf{Gx}}$

Consider the genomic relationship matrix ${\mathbf{G}} = {\mathbf{ZZ}}^{\prime } /k$ [13], where ${\mathbf{Z}}$ is a matrix of genotypes for $n$ animals and $m$ markers coded additively, and often “centered” locus-wise with reference either to base or to observed allele frequencies, and $k$ is a scale factor, typically the sum of heterozygosities at the markers. Weights for each locus can be introduced in the form ${\mathbf{G}} = {\mathbf{Z}}{\mathbf{D}}{\mathbf{Z}}^{\prime }$, and methods in this paper extend easily to this case. Aguilar et al. [14] presented efficient methods to compute ${\mathbf{G}}$. To compute products, it is more efficient to use ${\mathbf{G}}{\mathbf{x}} = {\mathbf{Z}}({\mathbf{Z}}^{\prime } {\mathbf{x}})/k$ (without explicitly forming ${\mathbf{G}}$) at a quadratic cost $2mn$ instead of the cubic cost of forming first ${\mathbf{G}}$ (cubic cost $mn^{2}$) to later compute ${\mathbf{Gx}}$ (quadratic cost $n^{2}$). The exception is when $n$ is small compared to $m$, in which case it is easier to compute and store ${\mathbf{G}}$.

Either of the matrix–vector products in ${\mathbf{G}}{\mathbf{x}} = {\mathbf{Z}}({\mathbf{Z}}^{\prime } {\mathbf{x}})/k$ can be programmed using public, already optimized, possibly parallel, subroutines such as DGEMV from BLAS [15]. Note that optimal contribution decisions are invariant to the choice of the reference allele (which results in the same ${\mathbf{G}}$) or to different estimates of base allelic frequencies used in ${\mathbf{Z}}$ and $k$, because changing assumed allele frequencies only scale and sum constants to ${\mathbf{Gx}}$ but the optimum is the same.

### Recalling the properties of the indirect computation of the matrix product ${\mathbf{Ax}}$

Vectors ${\mathbf{Ax}}$ (where ${\mathbf{x}}$ is any vector) can be quickly obtained following [4] based on the well-known fact that the sparse matrix ${\mathbf{A}}^{ - 1}$ is the product of an upper sparse triangular matrix by its transpose [16, 17]. The fast method is very handy to compute portions or functions of ${\mathbf{A}}$ wihout explicitly setting it up. For instance, extracting sections of ${\mathbf{A}}$ column-wise can be done by computing column $i$ as the product ${\mathbf{Ax}}$, where ${\mathbf{x}}$ contains 1 in position $i$ and 0 elsewhere. After only a single run, it also allows the computation of average relationships within groups of individuals $\overline{a} = {\mathbf{x^{\prime}Ax}}$ or between two groups of individuals $\overline{a} = {\mathbf{y^{\prime}Ax}}$, where ${\mathbf{x}}$ and ${\mathbf{y}}$ are vectors of individual contributions. On the contrary, setting up explicitly matrix ${\mathbf{A}}$ by the tabular rule is prohibitive because it involves a number of operations equal to the square of the number of individuals in the pedigree of candidates, which can be very large.

### Computation of the matrix product ${\mathbf{Hx}}$

Matrix ${\mathbf{H}}$ expands genomic information contained in ${\mathbf{G}}$ to ungenotyped individuals via pedigree relationships as follows [9, 12]:

$${\mathbf{H}} = {\mathbf{A}} + \left[ {\begin{array}{*{20}c} {{\mathbf{A}}_{12} {\mathbf{A}}_{22}^{ - 1} } & \mathbf{0} \\ \mathbf{0} & {\mathbf{I}} \\ \end{array} } \right]\left[ {\begin{array}{*{20}c} {\mathbf{I}} \\ {\mathbf{I}} \\ \end{array} } \right]\left[ {{\mathbf{G}} - {\mathbf{A}}_{22} } \right]\left[ {\begin{array}{*{20}c} {\mathbf{I}} & {\mathbf{I}} \\ \end{array} } \right]\left[ {\begin{array}{*{20}c} {{\mathbf{A}}_{22}^{ - 1} {\mathbf{A}}_{21} } & \mathbf{0} \\ \mathbf{0} & {\mathbf{I}} \\ \end{array} } \right]\varvec{ }$$

where subindexes 1 and 2 refer to ungenotyped and genotyped individuals, respectively.

The inverse of ${\mathbf{H}}$ is sparse and regularly used in single-step GBLUP. However, computing ${\mathbf{Hx}}$ is more demanding than any of the two previous cases ${\mathbf{Ax}}$ or ${\mathbf{Gx}}$, because it involves dense products and inverses that involve ${\mathbf{A}}_{22}$ and ${\mathbf{G}}$. The first purpose of our paper is to show this complexity and how this computation can be efficiently carried out.

An additional problem arises from the fact that the two terms forming ${\mathbf{H}}$, i.e., ${\mathbf{G}}$ and ${\mathbf{A}}$, should ideally refer to the same genetic base. Vitezica et al. [11] and Christensen et al. [18] suggested to compute ${\mathbf{G}}$ first using observed allele frequencies and then to convert it into matrix $\widetilde{{\mathbf{G}}}$, following metrics of pedigree base. The conversion principle was that the average of $\widetilde{{\mathbf{G}}}$ and its average diagonal should be equal to their counterparts in matrix ${\mathbf{A}}_{22}$. Then, $\widetilde{{\mathbf{G}}} = \alpha {\mathbf{J}} + \beta {\mathbf{G}}\varvec{ }$, where shift parameter $\alpha$ and scale parameter $\beta$ were obtained from four means: the average terms $\overline{{\mathbf{A}}}_{22}$ and $\overline{{\mathbf{G}}}$, and the average diagonal terms $\overline{d} \left( {{\mathbf{A}}_{22} } \right)$ and $\overline{d} \left( {\mathbf{G}} \right)$. Based on the two constraints, $\beta = \frac{{\overline{d} \left( {\mathbf{G}} \right) - \overline{{\mathbf{G}}} }}{{\overline{d} \left( {{\mathbf{A}}_{22} } \right) - \overline{{\mathbf{A}}}_{22} }}$ and $\alpha = \overline{{\mathbf{A}}}_{22} - \beta \overline{{\mathbf{G}}}$. This can be understood as correcting for drift of the overall mean ($\alpha$) and reduction in variance ($\beta$) [19]. If the genotyped population is large enough and mating is approximately at random, then average inbreeding (in either ${\mathbf{G}}$ or ${\mathbf{A}}_{22}$) is the average half relationships and $\beta \approx 1 - \frac{\alpha }{2}$. These coefficients can also be interpreted as $\alpha = 2F_{st}$ and $\beta = 1 - F_{st}$, where $F_{st}$ is a measure of differentiation from the more recent genotyped population in ${\mathbf{G}}$ to the base population of ${\mathbf{A}}_{22}$ [11, 19, 20].

However, considering the genomic base as the reference is preferable, i.e. modifying ${\mathbf{A}}$, not ${\mathbf{G}}$. Indeed, matrix ${\mathbf{A}}$ depends on pedigree recording and relies upon the assumption that pedigree founders are fully unrelated. This assumption can be removed using the metafounder approach [21], which postulates that the pedigree-based additive relationship between any pair of founders is equal to a positive parameter $\gamma$ (from 0 to 2) that summarizes the situation of the pedigree base in reference to the genomic base [10]. This parameter $\gamma$ can be estimated from genomic data [22], and represents the homozygosity across founders in the pedigree that would yield observed genomic relationships in ${\mathbf{G}}$, where ${\mathbf{G}}$ is computed as the cross-product ${\mathbf{G}} = {\mathbf{ZZ}}^{\prime } /\left( {2/m} \right)$ with ${\mathbf{Z}}$ containing {− 1, 0, 1} values. Furthermore, Garcia-Baccino et al. [22] showed that the value of $\gamma$ is relative to a theoretical genomic base that displays maximum variability at each marker locus (allelic frequencies 0.5), thus giving rise by drift to the pedigree base and to differentiation of frequencies in the genotyped population. Then, $\gamma$ is simply eight times the variance of the (unobserved) marker frequencies in the pedigree founders. In this context, they interpreted $\gamma$ as an $F_{\text{st}}$ index [23] and they proposed several estimation methods for parameter $\gamma$. The metafounder approach extends easily to several breeds or origins (e.g. genetic groups) by considering ${\varvec{\Gamma}}$, a matrix extension of $\gamma$, and this also provides an elegant solution to the problem of computing relationships including unknown parent groups [17], a case for which relationship is not a well-defined concept. As a result, we considered the metafounder approach to be adequate for the monitoring and management of genetic variability.

### Direct computation of matrix product ${\mathbf{Hx}}$

The following algorithms to compute ${\mathbf{Hx}}$ use the pedigree-based matrix ${\mathbf{A}}$ [24] and they are exactly the same when including metafounders in ${\mathbf{A}}^{{\left[\varvec{\varGamma}\right]}}$ [21].

Matrix ${\mathbf{H}}$ has the following components:

$${\mathbf{H}}_{11} = {\mathbf{A}}_{11} + {\mathbf{A}}_{12} {\mathbf{A}}_{22}^{ - 1} \left( {{\mathbf{G}} - {\mathbf{A}}_{22} } \right){\mathbf{A}}_{22}^{ - 1} {\mathbf{A}}_{21} ,$$

$${\mathbf{H}}_{12} = {\mathbf{A}}_{12} {\mathbf{A}}_{22}^{ - 1} {\mathbf{G}},$$

$${\mathbf{H}}_{21} = {\mathbf{GA}}_{22}^{ - 1} {\mathbf{A}}_{21} ,$$

$${\mathbf{H}}_{22} = {\mathbf{G}}.$$

Let $${\mathbf{y}} = {\mathbf{Hx}} = \left( {\begin{array}{*{20}c} {{\mathbf{y}}_{1} } \\ {{\mathbf{y}}_{2} } \\ \end{array} } \right) = \left( {\begin{array}{*{20}c} {{\mathbf{H}}_{11} } & {{\mathbf{H}}_{12} } \\ {{\mathbf{H}}_{21} } & {{\mathbf{H}}_{22} } \\ \end{array} } \right)\left( {\begin{array}{*{20}c} {{\mathbf{x}}_{1} } \\ {{\mathbf{x}}_{2} } \\ \end{array} } \right)$$ be the product of matrix ${\mathbf{H}}$ by any vector ${\mathbf{x}}$. The matrix expression of ${\mathbf{y}}_{2} = {\mathbf{H}}_{21} {\mathbf{x}}_{1} + {\mathbf{H}}_{22} {\mathbf{x}}_{2} = {\mathbf{GA}}_{22}^{ - 1} {\mathbf{A}}_{21} {\mathbf{x}}_{1} + {\mathbf{Gx}}_{2}$ is fairly simple compared to the expression of ${\mathbf{y}}_{1} = {\mathbf{H}}_{11} {\mathbf{x}}_{1} + {\mathbf{H}}_{12} {\mathbf{x}}_{2} = \left( {{\mathbf{A}}_{11} + {\mathbf{A}}_{12} {\mathbf{A}}_{22}^{ - 1} \left( {{\mathbf{G}} - {\mathbf{A}}_{22} } \right){\mathbf{A}}_{22}^{ - 1} {\mathbf{A}}_{21} } \right){\mathbf{x}}_{1} + {\mathbf{A}}_{12} {\mathbf{A}}_{22}^{ - 1} {\mathbf{Gx}}_{2}$ due to the complexity of ${\mathbf{H}}_{11}$. If ${\mathbf{w}}$ denotes a working vector, intermediate computations such as ${\mathbf{Gw}}$, ${\mathbf{Aw}}$ (indirect method) and ${\mathbf{A}}_{22}^{ - 1} {\mathbf{w}}$ (iterative or exact solving) are involved. The computation sequence that has to be carried out in order to obtain ${\mathbf{H}}_{11} {\mathbf{x}}_{1}$ is quite long. Fortunately, results can be obtained more efficiently by an indirect method as detailed below.

### An indirect computation of matrix product ${\mathbf{Hx}}$

The computation method is indirect for two reasons. First, because it uses the difference ${\mathbf{d}} = {\mathbf{y}} - {\mathbf{z}}$ between ${\mathbf{y}} = {\mathbf{Hx}}$ and ${\mathbf{z}} = {\mathbf{Ax}}$. Second, the method exploits the very simple expression of the inverse matrix ${\mathbf{H}}^{ - 1}$ [12, 25]:

$${\mathbf{H}}^{ - 1} = {\mathbf{A}}^{ - 1} + \left( {\begin{array}{ll} \mathbf{0} & \mathbf{0} \\ \mathbf{0} & {{\mathbf{G}}^{ - 1} - {\mathbf{A}}_{22}^{ - 1} } \\ \end{array} } \right),$$

so that

$${\mathbf{AH}}^{ - 1} = {\mathbf{I}} + {\mathbf{A}}\left( {\begin{array}{ll} \mathbf{0} & \mathbf{0} \\ \mathbf{0} & {{\mathbf{G}}^{ - 1} - {\mathbf{A}}_{22}^{ - 1} } \\ \end{array} } \right) = {\mathbf{I}} + \left( {\begin{array}{ll} \mathbf{0} & {{\mathbf{A}}_{12} \left( {{\mathbf{G}}^{ - 1} - {\mathbf{A}}_{22}^{ - 1} } \right)} \\ \mathbf{0} & {{\mathbf{A}}_{22} \left( {{\mathbf{G}}^{ - 1} - {\mathbf{A}}_{22}^{ - 1} } \right)} \\ \end{array} } \right).$$

To obtain ${\mathbf{d}}$, note that ${\mathbf{x}} = {\mathbf{H}}^{ - 1} {\mathbf{y}}$. Then:

1 $${\mathbf{z}} = {\mathbf{Ax}} = {\mathbf{AH}}^{ - 1} {\mathbf{y}} = {\mathbf{y}} + \left( {\begin{array}{*{20}c} \mathbf{0} & {{\mathbf{A}}_{12} \left( {{\mathbf{G}}^{ - 1} - {\mathbf{A}}_{22}^{ - 1} } \right)} \\ \mathbf{0} & {{\mathbf{A}}_{22} \left( {{\mathbf{G}}^{ - 1} - {\mathbf{A}}_{22}^{ - 1} } \right)} \\ \end{array} } \right){\mathbf{y}}$$

Consequently,

2 $${\mathbf{z}}_{2} = {\mathbf{y}}_{2} + {\mathbf{A}}_{22} \left( {{\mathbf{G}}^{ - 1} - {\mathbf{A}}_{22}^{ - 1} } \right){\mathbf{y}}_{2} ,$$

and

3 $${\mathbf{y}}_{2} = \left( {{\mathbf{I}} + {\mathbf{A}}_{22} \left( {{\mathbf{G}}^{ - 1} - {\mathbf{A}}_{22}^{ - 1} } \right)} \right)^{ - 1} \varvec{ }{\mathbf{z}}_{2} = {\mathbf{GA}}_{22}^{ - 1} {\mathbf{z}}_{2} .$$

Then, we obtain ${\mathbf{d}}_{2} = {\mathbf{y}}_{2} - {\mathbf{z}}_{2}$.

From Eq. (1), we obtain ${\mathbf{z}}_{1} = {\mathbf{y}}_{1} + {\mathbf{A}}_{12} \left( {{\mathbf{G}}^{ - 1} - {\mathbf{A}}_{22}^{ - 1} } \right){\mathbf{y}}_{2}$, whereas from Eq. (2) we obtain $\left( {{\mathbf{G}}^{ - 1} - {\mathbf{A}}_{22}^{ - 1} } \right){\mathbf{y}}_{2} = - {\mathbf{A}}_{22}^{ - 1} {\mathbf{d}}_{2}$, leading to:

4 $${\mathbf{d}}_{1} = {\mathbf{A}}_{12} {\mathbf{A}}_{22}^{ - 1} {\mathbf{d}}_{2} .$$

Finally, ${\mathbf{y}}_{1} = {\mathbf{z}}_{1} + {\mathbf{d}}_{1}$. Then, computing ${\mathbf{y}}_{1}$ through the indirect method is as simple as for ${\mathbf{y}}_{2}$, in total contrast with the direct method.

To summarize, in order to compute ${\mathbf{y}} = {\mathbf{Hx}}$:

1. Compute $${\mathbf{z}} = {\mathbf{Ax}}$$ using [4],
2. Compute $${\mathbf{y}}_{2} = {\mathbf{GA}}_{22}^{ - 1} {\mathbf{z}}_{2} = {\mathbf{G}}\left( {{\mathbf{A}}_{22}^{ - 1} {\mathbf{z}}_{2} } \right)$$,
3. Compute $${\mathbf{d}}_{2} = {\mathbf{y}}_{2} - {\mathbf{z}}_{2}$$,
4. Compute $${\mathbf{d}}_{1} = {\mathbf{A}}_{12} {\mathbf{A}}_{22}^{ - 1} {\mathbf{d}}_{2}$$,
5. Compute ${\mathbf{y}}_{1} = {\mathbf{z}}_{1} + {\mathbf{d}}_{1}$. This is the final step.

### Efficient solving

Product ${\mathbf{GA}}_{22}^{ - 1} {\mathbf{z}}_{2}$ can be obtained as ${\mathbf{G}}$ times vector ${\mathbf{A}}_{22}^{ - 1} {\mathbf{z}}_{2}$, using the fast method for ${\mathbf{Gx}}$ described before. The main numerical hurdle consists in solving linear equation systems that involve ${\mathbf{A}}_{22}$, a full matrix. Replacing these systems by others that involve matrix ${\mathbf{A}}^{11}$, a sparse matrix, is appropriate because ${\mathbf{A}}_{22}^{ - 1} = {\mathbf{A}}^{22} - {\mathbf{A}}^{21} \left( {{\mathbf{A}}^{11} } \right)^{ - 1} {\mathbf{A}}^{12}$. Furthermore, it is less time-consuming to restrict this equation to the genotyped individuals and their ancestors [26, 27]. If ${\mathbf{B}}$ denotes the relationship matrix corresponding to such a pedigree, then ${\mathbf{A}}_{22}^{ - 1} = {\mathbf{B}}^{22} - {\mathbf{B}}^{21} \left( {{\mathbf{B}}^{11} } \right)^{ - 1} {\mathbf{B}}^{12}$.

When programming, it can be handled as follows. For any working vector ${\mathbf{w}}$, let function $f\left( {\mathbf{w}} \right)$ return ${\mathbf{A}}_{22}^{ - 1} {\mathbf{w}}$ by extracting section 2 of vector $${\mathbf{B}}^{ - 1} \left( {\begin{array}{*{20}c} { - \left( {{\mathbf{B}}^{11} } \right)^{ - 1} {\mathbf{B}}^{12} {\mathbf{w}}} \\ {\mathbf{w}} \\ \end{array} } \right)$$, where products by ${\mathbf{B}}^{ - 1}$ and ${\mathbf{B}}^{12}$ can be obtained by the indirect method, and the linear equations involving matrix ${\mathbf{B}}^{11}$ can be solved by sparse matrix techniques [26, 28, 29]. Finally, Eq. (3) becomes ${\mathbf{y}}_{2} = {\mathbf{G}}f\left( {{\mathbf{z}}_{2} } \right)$ and Eq. (4) becomes ${\mathbf{d}}_{1} = {\mathbf{A}}_{12} f\left( {{\mathbf{d}}_{2} } \right)$ i.e., section 1 of vector $${\mathbf{A}}\left( {\begin{array}{*{20}c} \mathbf{0} \\ {f\left( {{\mathbf{d}}_{2} } \right)} \\ \end{array} } \right)$$.

### Computations in practical conditions

The indirect method can be used for monitoring and optimization diversity in large livestock populations: its implementation areas are briefly described in the “Appendix”. Usually, breeding organizations that are willing to control genetic variability consider at a given time an (possibly long) operational list. This list consists in male and/or female candidates for selection, possibly extended by the rest of the live population when generations are overlapping. Optimal contributions of candidates to the next generation, represented by vector ${\mathbf{x}}$, must be found, minimizing a function of the type $0.5{\mathbf{x}}'{\mathbf{Hx}} + {\mathbf{w}}'{\mathbf{x}}$ [22]. If all individuals in this operational list are genotyped, then computations are simple (at quadratic cost), restricted to the section of ${\mathbf{G}}$ individuals pertaining to the operational list. However, if some individuals in the operational list are not genotyped, computation of ${\mathbf{Hx}}$ vectors is needed. In this case, all genotyped animals add information to the full matrix ${\mathbf{H}}$ and the full ${\mathbf{G}}$ matrix should be used.

The fast indirect method is only used to compute (and possibly store) matrix ${\mathbf{H}}^{*}$, the section of ${\mathbf{H}}$ pertaining to the operational list. Afterwards, direct computations considering matrix ${\mathbf{H}}^{*}$ provide function derivatives and Lagrange multipliers when analytic optimisation methods are used [1, 3] or variations of functions for alternative contribution vectors when Monte-Carlo optimization is used [30, 31]. In the first case, a small number of configurations is considered before obtaining the optimal one, whereas this number can be very high for a Monte-Carlo method such as simulated annealing.

### Tayloring genomic relationship statistics to practitioners

In this section, we present elements to yield statistics in a scale that can be used by breeders. Genomic relationship coefficients derive from a statistical construction that has been basically developed for genomic evaluation purposes [13] although these coefficients are similar to marker-based relationships developed for conservation genetics [32]. Breeders and breeding organizations easily understand the output of research in the area of genetic evaluation, but understanding the concept of genomic relationships is more demanding. Practitioners are often puzzled by the unusual values of the genomic relationship coefficients (for instance negative genomic inbreeding, negative or very high relationships) in comparison with pedigree-based coefficients. This might deter breeders from implementing an effective genomic management of diversity.

A pragmatic compromise consists in optimization based on genomic relationships, possibly with metafounders, while the results (e.g. average inbreeding) are converted into more conventional scales before editing in output files. Conversion into conventional (pedigree-based) coefficients is carried out via a shift factor $\alpha^{conversion}$ and a scale factor $\beta^{conversion}$. We use the superscript “conversion” because these factors have not the same meaning as the $\alpha$ and $\beta$ in the section on “Computation of matrix product ${\mathbf{Hx}}$”: these are essentially operational factors. For instance, they cannot be interpreted as drift between pedigree founders and genotyped individuals in later generations [11]. These factors $\alpha^{conversion}$ and $\beta^{conversion}$ should be computed “once for all” based on a reference set of individuals that are genotyped before the effective start of genomic selection. Estimation forces equality of diagonals and overall means of ${\mathbf{G}}$ or ${\mathbf{H}}$ (computed with metafounders) and ${\mathbf{A}}$ (computed without metafounders), so that ${\mathbf{H}}^{converted} = \alpha^{conversion} {\mathbf{J}} + \beta^{conversion} {\mathbf{H}}$, and output files meet the familiar scale of probabilities of identity-by-descent from unrelated founders. First, stability of conversion factors is required to allow management and monitoring of genomic variability across cohorts over time, i.e. the average inbreeding in 2016 can be reliably compared to the average inbreeding in 2017.

Moreover, $\alpha^{conversion}$ and $\beta^{conversion}$ need to be estimated based on the animals genotyped before genomic selection proceeds. Otherwise, the shift factor $\alpha^{conversion}$ would be biased negatively. This can be predicted from Sonesson et al. [5], who showed that, in the case of genomic selection with pedigree management, the average pedigree-based relationship increases less than the average genomic relationship. Conversion is unbiased if the rates ($\Delta F$) of genomic inbreeding over time, either directly or based on converted values, are the same. At times $t$ and $t + 1$, the average genomic relationship coefficients are $\overline{h}_{t}$ and $\overline{h}_{t + 1}$ with conversion formula $\overline{h}_{t}^{converted} = \alpha^{conversion} + \beta^{conversion} \overline{h}_{t}$. If the asymptotic regime has already been reached, then the rate of inbreeding based on genomics is $\Delta F = \frac{{\overline{h}_{t} - \overline{h}_{t - 1} }}{{2 - \overline{h}_{t} }}$. If the rate of inbreeding is evaluated based on converted values, then $\Delta F^{converted} = \frac{{\beta^{conversion} \left( {\overline{h}_{t} - \overline{h}_{t - 1} } \right) }}{{2 - \alpha^{conversion} - \beta^{conversion} \overline{h}_{t} }}$. Both expressions are equal when $\beta^{conversion} = 1 - \alpha^{conversion} /2$, which is usually the case if Hardy–Weinberg equilibrium holds in the genotyped population for which $\alpha^{conversion}$ and $\beta^{conversion}$ have been estimated [19], i.e. if genotyping is at random or before genomic selection proceeds, but will possibly not hold if genotyped animals are selected based on genomic evaluation.

## Results

### Application to real data

Pedigree and genomic data from dairy goat and dairy sheep breeding programs were used. The French dairy goat breed Alpine uses an optimized selection program where the average conventional relationship is minimized for desired genetic gains [2, 30, 31]. Genomic selection is under study [33] and is planned in a near future. The Manech Tête Rousse, (blonde faced Manech), MTR dairy sheep breed belongs to the genetic improvement schemes in the French Western Pyrenees that are transitioning towards genomic selection [34]. Management of diversity is carried out within paternal grand-sire families. The Alpine SNP file consists in 2069 individuals genotyped for 46,687 SNPs by the SNP50 Bead chip (Illumina Inc., San Diego, CA, USA). These individuals represented all the progeny-tested males born since 1999, plus some favorably progeny-tested males born from 1985 to 1998, and 1200 females, born in 2008 and 2009, from 11 sires involved in a QTL detection design. The size of the operational list in **x** for producing young bucks in 2016 was 1135 (129 genotyped and 1006 ungenotyped): 44 genotyped male candidates, 769 ungenotyped female candidates (the reason why the ${\mathbf{Hx}}$ methodology was considered), 322 reference individuals (85 genotyped and 237 ungenotyped). The size of the pedigree file of the 3075 individuals under investigation (i.e. 2069 + 1135 − 129) plus their ancestors was 33,117. In this part of the whole Alpine population, pedigree recording was satisfactory and as a result, the pedigrees of the youngest individuals were 10 to 11 equivalent generations long, on average, and this is why tracing back the 3075 individuals yielded 30,000 more individuals. All the sires and maternal grand-sires of the ungenotyped individuals of the operational list were genotyped. Then, these males provided the connection between the 1006 ungenotyped individuals and the initial 2069 genotyped individuals. Sections of the ${\mathbf{A}}$ and ${\mathbf{H}}$ matrices corresponding to these animals were obtained.

The MTR dataset consists of 2108 genotyped rams born between 1999 and 2009, and 500,626 pedigree records, corresponding to the whole pedigree of the breed. Rams were genotyped with the OvineSNP50 Bead chip (Illumina Inc., San Diego, CA, USA). After applying filtering criteria [34], 38,997 SNPs were retained. Table 1 shows the number of genotyped rams and of females with all four grand-parents known (only these females are used as dams of rams) per year. With this MTR dataset, we were not able to carry out the same studies as in Alpine goats because neither of the 2108 genotyped individuals had genotyped sire, dam or grandparents, and they did not constitute a clear operational list since they already had offspring, i.e., they were not candidates to selection. In this case, we computed average relationships per year to assess robustness of these statistics to using either ${\mathbf{A}}$ or ${\mathbf{H}}$.

**Table 1:** Number of genotyped rams and females with four grandparents in each year of birth for the Manech Tête Rousse breed

Year of birth	Males genotyped	Females with four grandparents known	Total
1999	91	5434	5525
2000	132	5469	5601
2001	128	5579	5707
2002	139	5724	5863
2003	130	5927	6057
2004	117	5757	5874
2005	135	6064	6199
2006	125	6250	6375
2007	186	6154	6340
2008	545	6307	6852
2009	380	5515	5895
Total	2108	64,180	66,288

For both breeds, these datasets were not affected by genomic selection, which is planned only for the near future. The genotypic values in ${\mathbf{Z}}$ were coded as − 1, 0, 1 and ${\mathbf{G}}$ obtained as ${\mathbf{G}} = {\mathbf{ZZ}}'/\left( {m/2} \right)$ for $m$ SNPs [10, 21, 22]. The conventional relationship coefficients considered for constructing matrix ${\mathbf{H}}$ introduced a single metafounder with parameter $\gamma$, estimated by generalized least squares [22].

In both cases, using the indirect method and optimized computations of ${\mathbf{Gw}}$, ${\mathbf{Aw}}$ (by the indirect method) and ${\mathbf{A}}_{22}^{ - 1} {\mathbf{w}}$, computations are inexpensive, taking a few seconds on a laptop for any of the two datasets. To give a flavor of timing, in an Apple Macbook with 4 threads, computation of ${\mathbf{Gw}} = {\mathbf{Z}}\left( {{\mathbf{Z}}\prime {\mathbf{x}}} \right)/k$ ($2nm$ operations) with 5000 simulated animals and 50,000 simulated SNPs took 0.4 s, whereas computation of ${\mathbf{G}}$ itself ($n^{2} m$ operations) took 37 s.

### Results for the Alpine breed

Parameter $\gamma$ was estimated as 0.30. This means that the genetic variance in the pedigree base (the metafounder gene pool) was only $0.85 = 1 - \gamma /2$ times that in the conceptual genomic base [21].

**Fig. 1:**
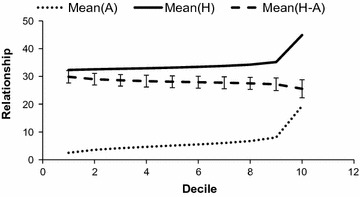
Average ${\mathbf{A}}$, average ${\mathbf{H}}$ and difference between ${\mathbf{H}}$ and ${\mathbf{A}}$ for the genotyped × genotyped section in the Alpine breed. Values were sorted by ascending relationship and divided into 10 deciles. Bars indicate standard deviation. Relationship coefficients are multiplied by 100

The terms of ${\mathbf{A}}_{22}$ (pedigree-based and not accounting for $\gamma$) were sorted by ascending order and classified into 10 groups of equal size (deciles). Figure 1 shows for each decile the average $a$, the average $h$, the average difference between both $a$ and $h$ and the standard deviation of the difference over replicates (after multiplication by 100 for clarity). Parameter $\gamma$ was estimated to be equal to 0.30, which explained the large average differences. The standard deviation of the difference was fairly constant irrespective of the decile considered. Differences in lower deciles were not less variable than in higher deciles: this meant that the relative impact of modifications was larger for lower pedigree-based coefficients. The order of magnitude of the standard deviation of the difference was 2. Expressed in usual terms (coancestry coefficient (%) in the classical pedigree base), this corresponded to $\frac{2 \times 0.5}{0.7} = 1.43$, a very small value. Finally, relationship modifications revealed by genotyping were substantial.

**Fig. 2:**
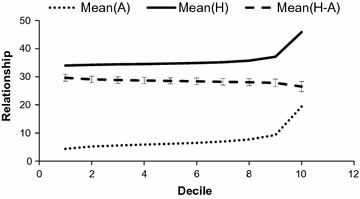
Average ${\mathbf{A}}$, average ${\mathbf{H}}$ and difference between ${\mathbf{H}}$ and ${\mathbf{A}}$ for the ungenotyped × genotyped section in the Alpine breed. Values were sorted by ascending relationship and divided into 10 deciles. Bars indicate standard deviation. Relationship coefficients are multiplied by 100

**Fig. 3:**
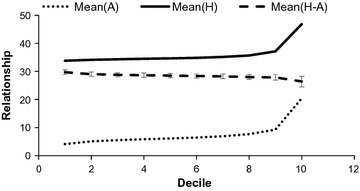
Average ${\mathbf{A}}$, average ${\mathbf{H}}$ and difference between ${\mathbf{H}}$ and ${\mathbf{A}}$ for the ungenotyped × ungenotyped section in the Alpine breed. Values were sorted by ascending relationship and divided into 10 deciles. Bars indicate standard deviation. Relationship coefficients are multiplied by 100

Deciles of ${\mathbf{A}}$ were constructed for the ungenotyped × genotyped section of the operational list (see Fig. 2) and for the ungenotyped × ungenotyped section (see Fig. 3). Basically, ${\mathbf{H}}$ **-**matrix genomic relationships involving ungenotyped individuals are estimated by regression and consequently, are intermediate between conventional relationships and true genomic relationships. The standard deviation of the difference between pedigree-based and genomic coefficients substantially decreased from 2 to 1.1 (Fig. 2) and 0.8 (Fig. 3) for the (ungenotyped, genotyped) and (ungenotyped, ungenotyped) sections, respectively. Thus, ${\mathbf{H}}$ estimations of genomic relationships for ungenotyped animals by regression yielded shrunken relationships, which were intermediate between genomic and pedigree-based coefficients in spite of the sires and maternal-grandsires of the ungenotyped individuals being genotyped. Consequently, if some candidates (*e.g.* dams of young males) are not genotyped in the future as in our operational list, the efficiency of the selection optimization will be affected in comparison with full genotyping. If the objective is to maximize genetic gain while constraining for genomic inbreeding rate [1], ungenotyped individuals with good estimated breeding values (EBV) will not be sufficiently selected because they cannot be shown to be “original”, leading to a loss for EBV. If the objective is to minimize inbreeding rate while constraining for genetic gain [2], as for the Alpine breed, favorable ungenotyped individuals will also be neglected, leading to a weaker minimization of inbreeding rate. Due to this partial genomic inbreeding control, targeted genetic gains are smaller than under full control.

From the 2069 genotyped individuals, 129 were in the operational list. The remaining 1940 genotyped individuals were reduced to 1500 or 1000 or 500 in order to check the effect of reducing the genomic information. These individuals were selected by considering the highest relationship with the 1006 ungenotyped individuals from the operational list to reduce the loss of genomic information. Three different values were obtained for the operational section of ${\mathbf{H}}$. These values were compared, term by term, with the values obtained with the complete matrix ${\mathbf{G}}$. When the size of the working ${\mathbf{G}}$ decreased, the average term of the operational section of ${\mathbf{H}}$ was lower than the reference average term (complete ${\mathbf{G}}$). The average difference was − 0.135, − 0.162 and − 0.183 (after multiplication by 100). Correspondingly, the standard deviation of the difference increased (0.180, 0.262 and 0.356). Thus, eliminating only 440 individuals out of 1940 (situation 1500) has already an impact, which indicates that it is important to use the largest possible ${\mathbf{G}}$, possibly including animals that are not candidates to selection.

**Fig. 4:**
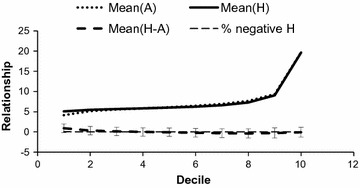
Statistics about the converted values in the Alpine breed. Values were sorted by ascending relationship and divided into 10 deciles. Bars indicate standard deviation. Relationship coefficients are multiplied by 100

Conversion of genomic relationships into pedigree-based coefficients for animals of the operational list provided the following results: shift factor $\alpha^{conversion} = - \,0.345$ and scale factor $\beta^{conversion} = 1.177$. Then, $\beta^{conversion}$ was very close to $1 - \alpha^{conversion} /2$, which would be the result obtained under Hardy–Weinberg equilibrium. The absence of negative bias on $\alpha^{conversion}$ might be due to the fact that data were obtained from a past breed history in conventional (not genomic) conditions of genetic evaluation, selection and management of diversity. Figure 4 shows the statistics about the converted values. Only a very small proportion of negative values was obtained in the lowest deciles (1–3).

### Results for the Manech Tête Rousse breed

Parameter $\gamma$ was estimated as 0.47. This means that the genetic variance in the pedigree base (the metafounder gene pool) was only 77% of that in the conceptual genomic base.

**Fig. 5:**
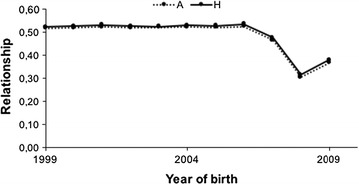
Evaluation of average relationships based on ${\mathbf{A}}$ and based on ${\mathbf{H}}$ in the Manech Tête Rousse breed

Figure 5 compares both alternative measures of overall relationship (both ${\mathbf{A}}$ and ${\mathbf{H}}$ include metafounders, thus they are comparable) and shows that, in general, both are very similar. The decrease in overall relationship observed from 2006 onwards is due to the larger number of rams genotyped (Table 1). Before this date, genotyped rams were only elite rams whereas from 2006 onwards, these were candidate rams, thus more diverse.

**Fig. 6:**
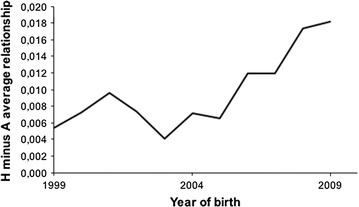
Mean value of the difference between ${\mathbf{H}}$ and ${\mathbf{A}}$ considering the 20 replicates in the Manech Tête Rousse breed

The mean value of the difference of relationships based on ${\mathbf{H}}$ or on ${\mathbf{A}}$ is represented in Fig. 6. Although very small, the trend seems to indicate that ${\mathbf{H}}$ detects more inbreeding than ${\mathbf{A}}$.

## Discussion

The analytic expressions of the relationship matrix ${\mathbf{H}}$ and its inverse are complex because the terms of ${\mathbf{H}}$ concerning the ungenotyped individuals are estimated by regression, conditionally on the observed genomic matrix ${\mathbf{G}}$. Managing or monitoring genomic variability when some of the individuals involved are not genotyped requires to be able to compute vectors ${\mathbf{Hx}}$. Consequently, a naive extension of the indirect method used for computing conventional vectors ${\mathbf{Ax}}$ to compute ${\mathbf{Hx}}$ provides tedious expressions. However, the analytic expressions are rather simple after considering the difference ${\mathbf{Hx}} - {\mathbf{Ax}}$ in two main computation steps. This makes the genomic indirect method easy to implement and very efficient. In this method, the conventional indirect method is used several times and the main computation hurdle is linked to dense matrices ${\mathbf{G}}$ and ${\mathbf{A}}_{22}$ (the pedigree-based counterpart of ${\mathbf{G}}$). If ${\mathbf{w}}$ denotes a working vector, computations of vectors ${\mathbf{Gw}}$ and ${\mathbf{A}}_{22}^{ - 1} {\mathbf{w}}$ are needed, but they can be carried out by efficient methods. Using ${\mathbf{G}}^{ - 1}$, or an approximation as in the algorithm for the proven and young animals (APY) algorithm [35], is possible but has no operational advantage because it results in a larger number of operations.

In spite of the above-mentioned methodological improvements, computing estimated genomic relationship coefficients when needed is quite demanding in terms of memory requirements and computation time. Although this does not pose a problem for national genetic evaluations, this might be a hurdle for some breeding companies that use local personal computers. First, all the genotyped individuals should be accounted for, even if they are little related or unrelated with the ungenotyped individuals under consideration (a fact confirmed by the study on the Alpine breed). This unfavorable finding can be puzzling at first sight but is quite natural because pedigree founders (typically nominally ‘unrelated’ individuals) exhibit substantial genomic relationships (the $\gamma$ parameter). Then, it is easy to infer that every member of the population pedigree is linked to the genotyped population, even if nominally (through pedigree) “unrelated”. Second, many runs of the genomic indirect method should be carried out if the size of the operational list involved in managing procedures is large. This is also the case if monitoring procedures aim at estimating the average genomic inbreeding per cohort: these averages require computing each individual coefficient by a specific run of the indirect method.

Converting genomic coefficients into pedigree-based coefficients, ideally through formulas that are established before starting genomic selection, was proposed and tested on the Alpine data. This might help breeders to really implement genomic management in parallel with genomic selection, a mind attitude imperiously needed [5]. In particular, if management continues to be based on pedigree relationships, the decrease in the actual (genomic) variability will be faster than its estimate based on pedigree, a warning signal for breeders.

## Conclusions

We presented efficient computation methods of products ${\mathbf{Hx}}$ for the single-step relationship matrices, which combine genotyped and ungenotyped individuals. Our methods are efficient and extend well to large datasets based on existing appropriate algorithms for computation of products ${\mathbf{Gw}}$ and ${\mathbf{A}}_{22}^{ - 1} {\mathbf{w}}$. These algorithms are useful for the management of genetic diversity in the genomic era.

## Appendix

### The scope of the ${\mathbf{Ax}}$ and ${\mathbf{Hx}}$ methodologies concerning genetic diversity

#### The scope of ${\mathbf{Ax}}$ methodology (no genotyping)

This method allows fast computation of statistics concerning the evolution of inbreeding and relationship coefficients over time (monitoring). Frequent implementations are computations of within and between cohorts (e.g. bulls born in the same year vs. cows born in the same year) average relationships. In the first case, the ${\mathbf{Ax}}$ methodology provides the average relationship very easily, e.g. within year of birth; in the second case, it provides the average coefficient for all possible pairs across the two cohorts.

The ${\mathbf{Ax}}$ method can also easily provide the average relationship of a group with a given individual. In all these cases, matrix ${\mathbf{A}}$ is never constructed, a useful property when very large commercial populations are considered.

Optimization of selection in nuclei and studs is a major field of implementation of breeding programs. Selection can be performed to maximize genetic gain while constraining the inbreeding rate or to minimize inbreeding rate while constraining the genetic gain. In both cases, the full ${\mathbf{A}}$ matrix pertaining to all candidates is needed, and possibly the vector of the average relationships of each candidate with a background group of non-candidate individuals. All these operations can be performed very easily with ${\mathbf{Ax}}$. Afterwards, based on this basic material, optimization can be performed by deterministic or Monte-Carlo methods to determine optimal contributions of each candidate.

When genomic selection has already started, but remains conventionally (pedigree) managed, an optimization akin to the previous one can be carried out for genotyping in selection nuclei, *i.e.*, choosing which young candidates (especially females) deserve to be genotyped, for a given genotyping investment.

Another implementation is the optimization of insemination in large commercial populations, in order to determine the contribution of each candidate and the resulting mating design. This requires the full table of relationships between male candidates and females to be known. It can be constructed after computing as many vectors ${\mathbf{Ax}}$ as the number of male candidates (generally a few in comparison with commercial population size).

#### The scope of ${\mathbf{Hx}}$ methodology (partial genotyping)

In genomic selection, the same operations as described above need to be done by the breeders: monitoring and handling by optimal contribution or similar methods. Ideally, this can be carried out using genomic relationships and the ${\mathbf{Gx}}$ methodology. However, it does happen that some individuals of interest are genotyped and others are not. The probability of such an event depends on the kind of implementation.

The methodology ${\mathbf{Hx}}$ is useful for monitoring the evolution of large commercial populations, where many females are ungenotyped. For selection nuclei with extensive genotyping, e.g. in pigs or poultry, where all animals are genotyped, it allows a full description of the evolution of the population including generations with old ungenotyped animals. For instance, it allows the comparison of the increase in inbreeding in pedigreed generations versus genotyped generations.

Optimizing genotyping will be permanently useful with open nuclei, where some females (e.g. rams’ dams) originate from commercial herds, are not necessarily genotyped, and for which the ${\mathbf{Hx}}$ methodology will be necessary.

Optimization of insemination in large commercial populations, with a large proportion of ungenotyped females will be always needed, which is an operation that requires the potential of the ${\mathbf{Hx}}$ methodology.
